# ‘Striking the Right Balance’ in Targeting PPAR*γ* in the Metabolic Syndrome: Novel Insights from Human Genetic Studies

**DOI:** 10.1155/2007/83593

**Published:** 2007-03-19

**Authors:** Mark Gurnell

**Affiliations:** Department of Medicine, University of Cambridge, Addenbrooke's Hospital, Hills Road, Cambridge CB2 2QQ, UK

## Abstract

At a time when the twin epidemics of obesity and type 2 diabetes threaten to engulf even the most well-resourced Western healthcare systems, the nuclear receptor peroxisome proliferator-activated receptor *γ* (PPAR*γ*) has emerged as a *bona fide* therapeutic target for treating human metabolic disease. The novel insulin-sensitizing antidiabetic thiazolidinediones (TZDs, e.g., rosiglitazone, pioglitazone), which are licensed for use in the treatment of type 2 diabetes, are high-affinity PPAR*γ* ligands, whose beneficial effects extend beyond improvement in glycaemic control to include amelioration of dyslipidaemia, lowering of blood pressure, and favourable modulation of macrophage lipid handling and inflammatory responses. However, a major drawback to the clinical use of exisiting TZDs is weight gain, reflecting both enhanced adipogenesis and fluid retention, neither of which is desirable in a population that is already overweight and prone to cardiovascular disease. Accordingly, the “search is on” to identify the next generation of PPAR*γ* modulators that will promote maximal clinical benefit by targeting specific facets of the metabolic syndrome (glucose intolerance/diabetes, dyslipidaemia, and hypertension), while simultaneously avoiding undesirable side effects of PPAR*γ* activation (e.g., weight gain). This paper outlines the important clinical and laboratory observations made in human subjects harboring genetic variations in PPAR*γ* that support such a therapeutic strategy.

## 1. INTRODUCTION

The health of a nation has long been recognized to be a function of its wealth. Traditionally, countries with limited resources have struggled to eradicate diseases that are often considered a thing of the past in so-called “developed” or “industrialized” nations. However, in recent years it has become clear that wealth does not always equate with good health. Indeed, we now face the very real possibility that in the first half of this century, average life expectancy in industrialized countries such as the US and UK will plateau or decline, despite continuing economic growth and prosperity [1]. The obesity epidemic, which is currently sweeping through “Western civilization,” is undoubtedly the single biggest factor behind this “unwanted reversal” [1]. Recent figures from the US reveal an alarming 75% increase in the prevalence of obesity over the past 25 years, such that a third of the population is now officially obese, that is to say, at least 20% heavier than their ideal weight [2]. Many Western European countries and Japan are not far behind. Obesity is a major risk factor for insulin resistance, type 2 diabetes mellitus (T2DM), hypertension, and dyslipidaemia (particularly hypertriglyceridaemia and low high-density lipoprotein cholesterol (HDL-C)); this cluster of medical sequelae is often grouped together under the umbrella term “metabolic syndrome,” and over the past decade the thresholds that must be met for the diagnosis of this entity have been progressively refined, culminating most recently in a consensus statement from the International Diabetes Federation (Table 1). Not surprisingly, subjects who meet the diagnostic criteria for this disorder are at significantly increased risk of atherosclerotic cardiovascular disease (reviewed in [3]).

So how can we arrest/reverse this apparently relentless march towards “metabolic meltdown”? The solution seems obvious: more effective obesity prevention and treatment. Limiting caloric intake and increasing energy expenditure to promote neutral (or in obese subjects negative) rather than positive energy balance is likely to yield enormous benefits at both the individual and population levels. Indeed, “lifestyle intervention” studies have already convincingly demonstrated that the risk of developing complications such as T2DM can be significantly reduced using such an approach [4, 5]. Unfortunately however, while this is a laudable goal, most clinicians know only too well that in practice it is very difficult to achieve/sustain, and hence attention has turned towards seeking novel therapies that are capable of ameliorating/reversing weight gain, insulin resistance, and their unwanted sequelae. Understanding the genes that are involved in maintaining metabolic homeostasis in the face of differing nutritional and environmental stresses is essential to the rational development of these strategies.

In recent years, a group of transcription factors belonging to the nuclear receptor superfamily has emerged as key players in the regulation of mammalian metabolism. Peroxisome proliferator-activated receptor *γ*(PPAR*γ*) is perhaps the best characterized of these so-called metabolic nuclear receptors, serving as it does to integrate the control of energy, glucose, and lipid homeostasis. The activity of PPAR*γ* is governed by the binding of small lipophilic ligands, principally fatty acids, derived from nutrition or metabolism [6, 7], and activation of the receptor is a critical step in the pathway to adipocyte differentiation and fat cell maturation. Hence, it is easy to envisage how chronic exposure to high levels of dietary PPAR*γ* ligands (provided in abundance in the Western diet) could promote the development of obesity, insulin resistance, and metabolic dysfunction, and why receptor modulation might offer a route to prevention/amelioration of these important cardiovascular risk factors. Indeed, drugs targeting PPAR*γ* activity (thiazolidinediones (TZDs), e.g., rosiglitazone, pioglitazone) are already in widespread clinical use as effective antidiabetic agents, enhancing insulin sensitivity, elevating high-density lipoprotein cholesterol (HDL-C) levels, and lowering blood pressure [8]. Importantly other studies have begun to examine whether these agents actually lower cardiovascular event rates [9], and if they are capable of reducing the risk of progression to overt T2DM in those with existing impaired glucose regulation [10].

Paradoxically however, TZDs actually promote weight gain rather than weight loss. A significant part of this increase can be attributed to enhanced adipogenesis, consistent with TZDs acting as high-affinity agonists for PPAR*γ* [11–13]. In addition, fluid retention and expansion of the extracellular compartment (possibly through altered renal sodium handling [14]) may contribute to weight gain in some patients, especially those with preexisting cardiac impairment [15]. Together, these observations raise an important question: is it possible to develop more selective PPAR*γ* modulators, with even greater potential to improve metabolic dysfunction, yet at the same time with reduced propensity to cause weight gain and fluid retention? Clearly, the answer to this question is dependent on the basic biology of PPAR*γ* and whether it proves possible to regulate receptor function in a tissue- and a target-gene-specific manner.

This paper summarizes the important contributions that human genetic studies have made to our understanding of the role of PPAR*γ* in the regulation of mammalian metabolic homeostasis, emphasizing the potential benefits and limitations that we can expect from more targeted approaches to modulating receptor function, and thus ensuring that in an era marked by an increasing prevalence of obesity, diabetes and cardiovascular disease, PPAR*γ* remains more of “a help” than “a hindrance.”

## 2. PPAR*γ*-STRUCTURE, FUNCTION, AND LIGAND REGULATION

The human nuclear receptor superfamily comprises 48 ligand-inducible transcription factors that respond to a variety of stimuli including steroid and thyroid hormones, vitamins, lipid metabolites, and xenobiotics. PPAR*γ* is the third member of a subdivision within the superfamily that also includes PPAR*α* and PPAR*δ* [25, 26]. Together, the PPARs function as key transcriptional regulators that govern metabolic homeostasis by serving as lipid sensors, responding to dietary fatty acids and their derivatives. However, each has a distinct pattern of tissue expression, and consistent with this, specific roles in the regulation of energy metabolism (reviewed in [25, 26]). The importance of these receptors in physiology and disease is evidenced by the fact that PPAR*α* and PPAR*γ* are the molecular targets for the lipid-lowering fibrate class of drugs and TZDs, respectively, while PPAR*δ* ligands are currently being developed in anticipation that they will offer a novel approach to tackling obesity and metabolic dysfunction through effects on energy expenditure, HDL-C metabolism, and macrophage inflammatory responses (reviewed in [26]).

**Figure 1:**
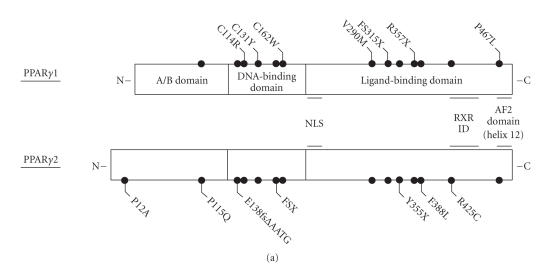

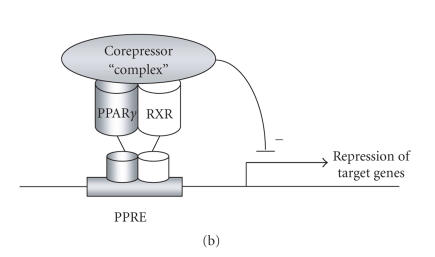

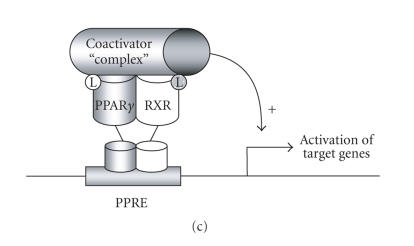
Structure function of PPAR*γ*. (a) Schematic representation of the three principal domains of PPAR*γ*, denoting the positions of several of the natural genetic variants that have been identified in the human receptor. Note that mutations and polymorphisms have been depicted based on the nomenclature (*γ*1 or *γ*2) used in the primary publication [16–23]. FSX denotes the mutation (A^553^ΔAAAiT)fs185(stop186); FS315X denotes the mutation (A^935^ΔC)fs312(stop315). (b) In the absence of exogenous ligand, PPAR*γ* recruits a corepressor complex to a subset of target genes (e.g., adipocyte glycerol kinase), thereby repressing basal transcription [24]. (c) Addition of ligand induces a conformational change in the receptor, which promotes corepressor release and coactivator recruitment. For other target genes (e.g., aP2), the receptor appears to be constitutively active even in the absence of exogenous ligand [24]. NLS denotes nuclear localization signal; RXR denotes retinoid X receptor; ID denotes interaction domain; AF2 denotes activation function 2; PPRE denotes PPAR response element.

Differential promoter usage, coupled with alternate splicing of the *PPARG* gene, generates two protein isoforms: PPAR*γ*2, expressed from a single *γ*2 promoter, contains an additional 28 N-terminal amino acids and is nearly adipose-specific; PPAR*γ*1, whose expression can be regulated by multiple (*γ*1, *γ*3, *γ*4) promoters, is more ubiquitously distributed [27–29]. Like other nuclear receptors, PPAR*γ* exhibits a modular structure consisting of distinct functional domains: the N-terminal A/B domain harbors a ligand-independent transcriptional activation function (AF1), which is stronger for the *γ*2 than *γ*1 isoform; the central DNA-binding domain, containing two zinc finger motifs, facilitates interaction with specific binding sites (PPAR response elements (PPREs)) in target gene promoters; the larger C-terminal domain mediates ligand-binding, heterodimerization with the retinoid X receptor (RXR), and contains a powerful ligand-dependent activation (AF2) function (Figure 1(a)).

Initially, PPAR*γ* was considered to be a constitutively active receptor, recruiting transcriptional coactivators (e.g., steroid receptor coactivator-1 (SRC-1)) to classical target genes (e.g., adipocyte protein 2 (aP2)) even in the absence of ligand. More recently however, Guan et al. have shown that the unliganded PPAR*γ*/RXR heterodimer can actively silence a subset of genes (e.g., adipocyte glycerol kinase (GyK)), in a manner analogous to that seen with the thyroid hormone (TR) and retinoic acid (RAR) receptors [24] (Figure 1(b)). Transcriptional silencing is mediated through recruitment of a multiprotein corepressor complex, containing either NCoR (nuclear receptor corepressor) or SMRT (silencing mediator of retinoic acid and thyroid receptors), together with histone-modifying enzymes (e.g., histone deacetylase 3 (HDAC 3)), which condense chromatin structure, thus impeding gene transcription. In contrast, binding of cognate or exogenous ligand(s) induces a conformational change in the heterodimer such that it now dissociates from any bound corepressor proteins and instead recruits a coactivator complex, containing histone acetyltransferases (e.g., CREB-binding protein (CBP)), which relaxes the chromatin structure so as to permit greater levels of gene transcription (Figure 1(c)).

A variety of putative endogenous activators has been described for PPAR*γ*, including fatty acids, eicosanoids, and derivatives of oxidized low-density lipoproteins [30]. The prostaglandin J2 derivative 15-deoxy-Δ^12,14^-PGJ2 is also capable of activating PPAR*γ* *in vitro*, although it is doubtful as to whether it exists at sufficient concentrations *in vivo* to serve as a physiological ligand. Recently, Tzameli et al. have reported the existence of an as yet undefined ligand(s) that is produced transiently during adipocyte differentiation [31].

## 3. PPAR*γ*-A KEY THERAPEUTIC TARGET IN THE HUMAN METABOLIC SYNDROME

Patients with the metabolic syndrome typically require a “cocktail of drugs” to treat the individual components of the disorder and its associated atherosclerotic complications (e.g., oral hypoglycaemic agents, insulin, statins, fibrates, antihypertensives, aspirin, etc.). Unfortunately, many of these drugs confer little benefit in terms of correcting the underlying metabolic disturbance, and indeed some even exacerbate the situation, for example, insulin-induced weight gain. Not surprisingly then, compliance with these complex treatment regimens is often poor.

In contrast, drugs that target PPAR*γ* appear, at least in theory, to offer an attractive and perhaps more logical approach to treating the metabolic syndrome, by virtue of their ability to ameliorate insulin resistance and other facets of the condition [8]. Set against this however is the well-documented increase in body weight that is observed with currently available TZDs [8]. It is these observations that have led scientists and clinicians alike to ask whether it is possible to retain/enhance the metabolic benefits of PPAR*γ* activation, yet at the same time minimize undesirable side effects. The following sections outline the human genetic evidence that supports such a strategy, with specific reference to each of the key components of the metabolic syndrome.

### 3.1. PPAR*γ* and adipogenesis

*in vitro* studies suggest that PPAR*γ* is the ultimate effector of adipogenesis in a transcriptional cascade that also involves members of the C/EBP transcription factor family [32]. Modulation of PPAR*γ* expression and/or action in rodent cell lines has conclusively shown that the receptor is both essential and, in the presence of PPAR*γ* agonists, is sufficient for adipogenesis [33]. Consonant with this, PPAR*γ* knockout mice fail to develop adipose tissue [34–36], while their heterozygous counterparts have reduced fat depots [36]. Studies in human tissues point to a similar critical role for PPAR*γ* in the regulation of adipogenesis. Exposure of cultured primary human preadipocytes to PPAR*γ* activators (e.g., TZDs) induces their differentiation [32], while both chemical and biological receptor antagonists efficiently block this process [37].

It comes as no surprise then to learn that human subjects treated with synthetic PPAR*γ* agonists (e.g., rosiglitazone, pioglitazone) gain weight through enhanced adipogenesis [8]. Despite this, metabolic function in the majority of TZD recipients improves. This apparent TZD paradox undoubtedly reflects the ability of these agents to modify adipocyte function and free fatty acid storage in a favorable manner that promotes insulin sensitization; however, it may also be dependent, at least in part, on PPAR*γ* activation mediating depot-specific rather than global changes in adipogenesis. For example, it is notable that the increase in fat mass observed in type 2 diabetics treated with TZDs is not uniformly distributed, with a tendency to accumulate subcutaneous (e.g., limb/gluteal) fat, whereas visceral adipose tissue volume is reduced or unchanged (reviewed in detail in [38]). Consistent with this, preadipocytes isolated from subcutaneous abdominal adipose tissue have been shown in some (although not all) studies to differentiate more readily in response to TZDs than cells from visceral depots taken from the same subjects [39].

#### 3.1.1. Gain- and loss-of-function mutations

With PPAR*γ* agonists promoting adipogenesis, it would seem reasonable to speculate that gain-of-function PPAR*γ* mutations should increase body fat mass. Ristow et al. have provided support for this hypothesis, with the identification of four morbidly obese (BMI 37.9 to 47.2 kg/m^2^) German subjects, all of whom harbored a gain-of-function mutation (Pro115Gln PPAR*γ*2) within the N-terminal domain of the receptor [40]. The transcriptional activity of PPAR*γ* is subject to modification through phosphorylation of a serine residue at codon 114 [41, 42], and mutation of the adjacent proline was shown to interfere with this process, resulting in a receptor with constitutive transcriptional activity and enhanced adipogenic potential [40]. Subsequently however, a fifth subject, with only a mildly elevated BMI (28.5 kg/m^2^), was found to carry the same amino acid substitution, which is in marked contrast to the findings of the original study [43]. Thus, for now the significance of this particular genetic variant remains unclear, and further mutation carriers must be identified to confirm whether Pro115Gln does indeed predispose to obesity and, if so, whether there is a depot-specific pattern to the accretion of adipose tissue.

In contrast, there is now a compelling body of data from the study of human subjects with loss-of-function mutations in PPAR*γ* to confirm a pivotal role for this receptor in human adipogenesis. To date, twelve different heterozygous mutations (missense, nonsense, and frameshift) have been identified within the DNA- (DBD) and ligand-binding (LBD) domains of the receptor (Figure 1(a)) [16–23], with functional studies, where available, confirming that the mutant receptors are transcriptionally impaired. In keeping with their dominant mode of inheritance, several of the mutants have also been shown to be capable of inhibiting the activity of their wild-type counterpart in a dominant negative manner, reflecting either aberrant corepressor recruitment to DNA-bound mutant receptors [16, 44], or transcriptional interference through coactivator sequestration by DNA-binding deficient mutants [23]. In contrast, other mutants appear to lack dominant negative activity, with the clinical phenotype purported to be a consequence of haploinsufficiency [18, 20–22]. In keeping with the latter, Al-Shali et al. have recently identified a kindred harboring a novel heterozygous A > G mutation at position −14 within intron B of *PPARG* (upstream of exon 1), which reduces promoter activity of the PPAR*γ*4 isoform [45]. This mutation cosegregated with a phenotype of partial lipodystrophy and metabolic dysfunction similar to that observed in subjects harboring loss-of-function mutations within the DBD or LBD [45].

**Figure 2:**
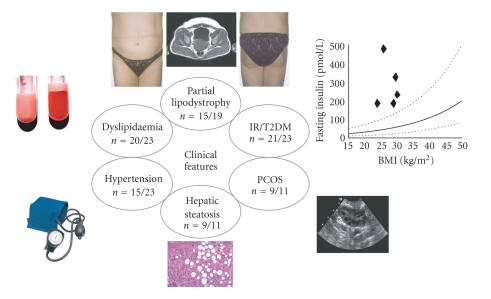
Clinical features exhibited by adult subjects harboring loss-of-function mutations in human PPAR*γ*. For each parameter shown, the numerator denotes the reported number of affected individuals, and the denominator denotes the number of subjects for whom relevant information is available.

Together, these reports describe more than twenty adult subjects, the majority of whom exhibit a stereotyped pattern of partial lipodystrophy, in which subcutaneous fat is diminished in the limbs and gluteal region, while being preserved/increased in the subcutaneous and visceral abdominal depots (Figure 2) [16–23]. Some phenotypic differences have been observed with facial and neck adipose tissues, which were reported to be increased in individuals from two kindreds, but normal or reduced in most other cases [16–23]. These findings are again strongly suggestive of a depot-specific role for PPAR*γ* in human adipogenesis, and complement the observations made in diabetic subjects receiving TZD treatment. Clearly one challenge is to understand why visceral adipose tissue appears relatively refractory to PPAR*γ* regulation despite expressing comparable levels of receptor to its subcutaneous counterpart. Studies of fat biopsies from different depots in PPAR*γ* mutation carriers might offer a unique route to addressing this important question.

Interestingly, a transgenic knockin mouse model based on the human Pro467Leu mutation (Pro465Leu) has recently been reported by two independent groups [46, 47]. Heterozygous *Pparg^P465L/+^* mice have normal total adipose tissue weight, but exhibit reduced intra-abdominal fat mass and increased extra-abdominal subcutaneous fat compared to wild-type (WT) animals, that is, altered body fat distribution, but in a manner which is quite distinct from that observed in human subjects. In addition, unlike their human counterparts, the *Pparg^P465L/+^* mice were also insulin-sensitive. These findings initially raised concerns as to the suitability of using rodent models to explore the consequences of loss-of-function mutations in human PPAR*γ*. Importantly however, in the model of Gray et al., expression of the P465L mutant on a hyperphagic ob/ob background grossly exacerbated the insulin resistance and metabolic disturbances associated with leptin deficiency, despite reducing whole body adiposity and adipocyte size [47]. Thus, in the mouse coexistence of the P465L PPAR*γ* mutation and the leptin-deficient state creates a mismatch between adipose tissue expandability and energy availability, thereby unmasking the deleterious effects of PPAR*γ* mutations on carbohydrate metabolism and recapitulating the clinical phenotype observed in human subjects.

#### 3.1.2. Polymorphisms

The most prevalent human PPAR*γ* genetic variant reported to date is the Pro12Ala polymorphism, substituting alanine for proline at codon 12 in the unique PPAR*γ*2 amino-terminal domain [48]. The allelic frequency of the Ala variant differs quite markedly depending on the study population, ranging from 1% to 23% [49]. In functional assays, Ala12-PPAR*γ* exhibits reduced binding to DNA and modest impairment in target gene transactivation in both the absence and presence of PPAR*γ* agonists [48]. An association with lower BMI in the primary study appeared to suggest a corresponding genotype-phenotype correlation, and led to the hypothesis that improved insulin sensitivity might be accounted for entirely by changes in adiposity [48]. However, numerous subsequent cross-sectional studies have yielded conflicting results, demonstrating either no difference [50] or a modestly greater BMI [51] in carriers of the Ala allele. In an attempt to resolve this issue, Masud and Ye completed a meta-analysis using data from 30 independent studies with a total of 19 136 subjects [52]. They concluded that in the samples with a mean BMI value ≥27 kg/m^2^, Ala12 allele carriers had a significantly higher BMI than noncarriers, whereas no difference was detected in the samples with a BMI value <27 kg/m^2^. A further analysis using data from publications in which BMI for the three genotype groups (i.e., Pro/Pro, Pro/Ala and Ala/Ala) were presented separately revealed that the Ala12 homozygotes had significantly higher BMI than heterozygotes and Pro12 homozygotes [52].

Importantly, the effects of the Ala allele have recently been shown to be subject to modification by other genetic and environmental factors, and indeed this may in part explain the apparently discordant results of the studies reported hitherto. For example, variations in dietary polyunsaturated fat versus saturated fat intake appear to influence BMI in carriers of the Ala variant [53]. In the Quebec Family Study, carriers of the Pro12 allele had lower BMI, waist circumference and fat mass (both subcutaneous and visceral) at baseline, but responded to an increase in dietary fat with a gradual increase in BMI and waist circumference, an effect which was not observed in their Ala counterparts [54]. Together, these and other studies support the notion of gene-nutrient interaction at the PPAR*γ* locus.

### 3.2. PPAR*γ* and insulin sensitivity

#### 3.2.1. Genetic evidence for a link

Several lines of evidence point to a link between the level of PPAR*γ* transcriptional activity and insulin sensitivity: (1) the *in vitro* binding affinities of TZD and non-TZD PPAR*γ* ligands correlate closely with their *in vivo* potencies as insulin sensitizers [11, 55]; (2) RXR ligands, which can activate the PPAR*γ*-RXR heterodimer, also exhibit insulin-sensitizing effects in rodents [56]; (3) mice exhibiting enhanced PPAR*γ* activity, due to a mutation at serine 112 (serine 114 in human PPAR*γ*2), which results in a constitutively more active receptor (through inhibition of phosphorylation), are protected from obesity-associated insulin resistance [57]; (4) mice lacking PPAR*γ* in fat, muscle, or liver are predisposed to developing insulin resistance [58–61].

Importantly, studies of human PPAR*γ* genetic variants have provided independent validation of the pharmacological and animal data. For example, severe insulin resistance (with or without overt T2DM) has proved to be a remarkably consistent finding in subjects with loss-of-function PPAR*γ* mutations, being evident even in early childhood in affected individuals (Figure 2) [16–23]. Equally impressive has been the finding that of more than 40 different reported associations of genetic variation and population risk to T2DM, Pro12Ala has emerged as the most widely reproduced [62]. The Ala allele is protective against the risk of developing T2DM, and it has been estimated that the global prevalence of T2DM would be ∼25% lower simply by virtue of everybody carrying one or more copies of the Ala allele [49, 62, 63], implying that PPAR*γ* is perhaps the single most important “diabetogene” identified to date.

In light of the findings with Pro12Ala, several groups have sought to determine whether other single-nucleotide polymorphisms (SNPs) within PPAR*γ* might also influence T2DM risk at a population level. In a study of ∼4000 Asian subjects, a link with a second polymorphism C1431T (for which the presence of a T allele conferred a reduced diabetes risk when compared with CC homozygotes (OR = 0.73, *P* = .011)) has been reported [64]. Other workers have taken analysis of this genetic variant further, establishing it to be in tight allelic disequilibrium with the Ala12 variant in a separate study population (70% of all Ala carriers also carried the C1431T polymorphism) [65]. Having genotyped individuals from three separate cohorts (1997 subjects with T2DM, 2444 nondiabetic children, and 1061 middle-aged controls—all from a similar area in Tayside, Scotland) for the *PPARG* Pro12Ala and C1431T polymorphisms, they concluded that the Ala12 variant was underrepresented in the T2DM population when compared with similarly aged nondiabetic adults (OR = 0.74, *P* = .0006). The 1431T variant was also underrepresented in the T2DM versus adult population. Intriguingly however, when the Ala12 variant was on a haplotype not bearing the 1431T variant, it conferred greater protection (OR = 0.66, *P* = .003); in contrast, when it was present in haplotypes containing the 1431T variant (70% of Ala12 carriers), this protection was absent (OR = 0.99, *P* = .94). Further studies are awaited with interest.

Thus, it is clear that the relationship between PPAR*γ* activity and insulin sensitivity in humans is complex, with evidence for a gene dosage effect, which is subject to modification by other genetic and environmental factors.

#### 3.2.2. Mechanisms of action

*Adipose tissue*

Given its high level of expression in adipose tissue and its pivotal role in adipogenesis, it is likely that receptor activation in adipocytes contributes significantly to the clinical efficacy of PPAR*γ* ligands in ameliorating insulin resistance. Consistent with this, mice lacking adipose tissue have been shown to be refractory to the antidiabetic effects of TZDs [66], while adipose-specific deletion of PPAR*γ* (which is associated with progressive lipodystrophy) predisposes mice to hepatic steatosis, and high-fat feeding-induced skeletal muscle insulin resistance [58]. In addition, because PPAR*γ*2 is virtually exclusively expressed in fat cells, any metabolic effects of the Pro12Ala polymorphism, including those on glucose homeostasis, are likely to be secondary to alterations in adipose tissue metabolism. Several mechanisms have been advanced to explain how modulating PPAR*γ* activity in fat benefits whole-body insulin sensitivity.

*(i) Regulation of free fatty acid flux in adipocytes*

Circulating levels of free fatty acids (FFAs) are a major determinant of insulin sensitivity [38]. Several studies have shown that the antidiabetic efficacy of TZDs correlates with their ability to lower circulating FFA levels [38]. Murine and cellular studies indicate that PPAR*γ* activation in adipose tissue may exert coordinated effects on FFA flux (promoting uptake/trapping, while simultaneously impairing release), through the regulation of a panel of genes involved in FFA metabolism: adipocyte lipoprotein lipase (LPL) expression is upregulated in response to TZD treatment, thereby potentially enhancing release of FFAs from circulating lipoproteins [67]; simultaneous upregulation of FFA transporters such as CD36 and FATP (fatty acid transport protein) on the adipocyte surface facilitates their uptake [68]; TZDs may also reduce FFA efflux from adipocytes through enhanced expression of genes that promote their storage in the form of triglycerides (e.g., glycerol kinase directs the synthesis of glycerol-3-phosphate directly from glycerol; phosphoenolpyruvate carboxykinase permits the utilization of pyruvate to form the glycerol backbone for triglyceride synthesis) [69, 70]. If similar effects on FFA uptake and trapping are observed in human adipocytes, then treatment with TZDs and other PPAR*γ* activators is likely to promote the safe storage of FFAs in adipose tissue, and prevent “ectopic” deposition in other sites such as liver and skeletal muscle, where they are capable of inducing “lipotoxicity.” Observations in human subjects with genetic variations in PPAR*γ* are consistent with this hypothesis. For example, it appears that even the existing residual adipose tissue depots in individuals with loss-of-function mutations in PPAR*γ* are dysfunctional, resulting in exposure of skeletal muscle and liver to unregulated fatty acid fluxes, with consequent impairment of insulin action at these sites [19]. In addition, there is evidence that the Pro12Ala polymorphism facilitates insulin-mediated suppression of lipolysis, hence decreasing FFA release [49]. It is worth noting however that others have failed to detect any relationship between circulating FFA levels and Pro12Ala status [71].

*(ii) Modulation of adipokine release*

In addition to regulating circulating FFA levels, adipocytes also serve as a rich source of signalling molecules (e.g., leptin, adiponectin, tumour necrosis factor-*α* (TNF*α*), and resistin), many of which have far-reaching metabolic effects in other tissues. Collectively these adipocyte-derived hormones are referred to as adipokines, and several have been identified as targets for transcriptional regulation by PPAR*γ*. In general, TZDs and other PPAR*γ* agonists enhance the expression of adipokines that facilitate insulin action while simultaneously suppressing those which are antagonistic, thereby altering the profile of adipocyte gene expression in a manner that promotes insulin sensitization. For example, activation of PPAR*γ* inhibits the expression of TNF*α*, resistin, and retinol-binding protein 4 (RBP4), all of which are associated with insulin resistance [72–74]. In contrast, adiponectin gene expression is increased following TZD treatment, thereby promoting fatty acid oxidation and insulin sensitivity in muscle and liver [75]. Circulating adiponectin levels have been shown to correlate closely with insulin sensitivity, and inversely with fat mass (especially visceral adiposity) [76], suggesting that this adipokine may represent a critical link between PPAR*γ* activation and insulin sensitization [75, 76]. Consonant with this, circulating adiponectin levels have been shown to be dramatically reduced in individuals harboring loss-of-function PPAR*γ* mutations when compared with healthy controls [77, 78]. In contrast, to date, no definitive correlation between the Pro12Ala polymorphism and adipokine release has been established, with existing studies providing conflicting results.

*(iii) Promotion of glucose uptake into adipocytes*

There is evidence to suggest that PPAR*γ* is also capable of directly modulating the insulin signal transduction pathway in adipose tissue. The GLUT4 (insulin-dependent) transporter is a key modulator of glucose disposal in both muscle and fat. Binding of insulin to its tyrosine kinase receptor engages a cascade of intracellular phosphorylation events, including activation of phosphatidylinositol-3-OH kinase (PI(3)K) and other downstream kinases, which promote trafficking of GLUT4 containing vesicles to the plasma membrane. A second pathway, which involves a distinct group of signalling molecules including the c-Cbl protooncogene product and CAP (c-Cbl-associated protein), acts in concert to augment this process. Several groups have shown that PPAR*γ* activation in adipose tissue can influence insulin signalling at various points in these pathways, for example, through upregulation of insulin receptor substrates-1 and -2 (IRS-1, IRS-2) [79, 80], the p85 subunit of PI(3)K [81], and CAP [82, 83]—all of which might be predicted to enhance GLUT4 activity. Increased glucose uptake into adipocytes contributes to whole-body glucose disposal, and provides important substrate for triglyceride synthesis.

*(iv) Regulation of adipocyte 11*β*-hydroxysteroid dehydrogenase type 1 activity*

Prolonged exposure to hypercortisolaemia, as occurs in subjects with Cushing's syndrome, is associated with many features of the metabolic syndrome (visceral obesity, glucose intolerance, hypertension, and dyslipidaemia). While circulating cortisol levels in ordinary obese non-Cushingoid individuals are normal (if not slightly reduced), there is evidence to suggest that local regeneration of cortisol within adipose tissue could contribute to the development of insulin resistance in the setting of visceral obesity [84]. 11*β*-hydroxysteroid dehydrogenase type 1 (11*β*-HSD1) directs the production of active cortisol from inactive cortisone in liver and fat, thereby facilitating cortisol-induced adipocyte differentiation. In keeping with this, adipose-specific overexpression of 11*β*-HSD1 in transgenic mice induced a phenotype of insulin resistance and central obesity [85]. PPAR*γ* ligands have been shown to downregulate adipocyte 11*β*-HSD1 expression and activity [86], and the subsequent modulation of glucocorticoid-induced gene expression may conceivably contribute to their insulin sensitizing actions. Studies of 11*β*-HSD1 activity in adipose tissue from subjects with loss-of-function mutations in PPAR*γ* should provide a unique opportunity to examine the role of PPAR*γ* in regulating human 11*β*-HSD1 function.

*Skeletal muscle and liver*

Maintenance of normal glucose homeostasis is critically dependent on retention of insulin sensitivity in key target tissues including liver and skeletal muscle. In addition to the beneficial effects of lowering circulating FFA levels and inducing a more favorable adipokine milieu to promote insulin sensitivity, there is some evidence to suggest that PPAR*γ* activation at both of these sites might directly influence glucose and lipid homeostasis. For example, TZDs have been reported to facilitate insulin-stimulated glucose uptake in cultured human skeletal muscle cells, by enhancing insulin-stimulated PI(3)K activity and GLUT4 translocation [87, 88]. Thus, while skeletal muscle expresses relatively low levels of PPAR*γ* protein when compared with adipose tissue, its dominant role in insulin-mediated glucose disposal suggests that PPAR*γ* activation at this site may contribute significantly to the glucose lowering effect of TZD treatment. Unfortunately, to date attempts to resolve this issue using animal models of muscle-specific PPAR*γ* deletion have proved unsuccessful with two separate groups reporting conflicting findings [59, 60]. Similarly, it remains to be seen whether activation of PPAR*γ* in human liver benefits or impairs metabolic function, with further studies needed to clarify its role in the regulation of hepatic gluconeogenesis and susceptibility to hepatic steatosis.

### 3.3. PPAR*γ* and lipid homeostasis

As might be predicted for a group of drugs that improve insulin sensitivity, TZDs raise HDL cholesterol in the majority of treated diabetics (typically by 5%–10% ) [8]. Intriguingly however, their effects on hypertriglyceridaemia have been somewhat more variable, with reductions in triglyceride levels observed more often with pioglitazone than rosiglitazone. One hypothesis that has been advanced to explain this apparent discrepancy is that pioglitazone may also be acting as a partial PPAR*α* agonist (akin to a fibrate), while at the doses used in clinical practice rosiglitazone retains pure *γ*-agonist activity [89]. However, data on mechanisms underlying the effects of TZDs on lipids in humans is limited and, moreover, caution needs to be exercised when attempting to extrapolate from animal studies, given the significant species-specific differences that exist in lipoprotein metabolism.

To date, virtually all subjects with loss-of-function mutations in PPAR*γ* have exhibited hypertriglyceridaemia and/or low HDL levels, with relatively unremarkable LDL cholesterols [16–23] (Figure 2). It remains unclear however, as to whether these abnormalities are simply a “metabolic consequence” of severe insulin resistance *per se*, or whether they indicate an additive and independent effect of dysfunctional PPAR*γ* signalling in relation to lipoprotein metabolism. Further studies of the reverse cholesterol transport pathway in monocyte-derived macrophages from these subjects may help to address this important issue.

Although there is an extensive body of data concerning the potential effects of the Pro12Ala polymorphism on glycaemic control, there are relatively few studies focusing on its consequences for lipid homeostasis. Moreover, given the potential confounding effect of insulin resistance, cohort selection (particularly with respect to diabetic status and/or BMI) is critical when trying to identify a specific independent link. Accepting these limitations, there is some evidence to suggest that the Ala allele may confer benefits for HDL metabolism. For example, in the original study of Deeb et al., higher HDL cholesterol (and lower triglyceride) levels were observed among elderly subjects with the Ala/Ala genotype compared with Pro/Ala and Pro/Pro genotypes [48]. A similar association has been described in over 4000 Singapore Asians whose genotype was analyzed as a dichotomous variable (i.e., presence or absence of the Ala variant), and in whom Ala allele carriers had significantly higher HDL cholesterol compared with Pro/Pro homozygotes [64]. However, other groups have reported conflicting findings, with some detecting an association of lower HDL cholesterol levels with the presence of the Ala allele [50].

### 3.4. PPAR*γ* and blood pressure regulation

Hypertension has been reported in a significant proportion of subjects harboring PPAR*γ* mutations [16–23]. While this is not unexpected, given the well-recognized associations of insulin resistance and T2DM with hypertension, it is noteworthy that in some cases the hypertension has been of an unusually early onset and severity [16, 19, 23]. Indeed, on occasion it has been the dominant clinical feature, manifesting even in the absence of diabetes and its associated microvascular complications. In contrast, TZD therapy is associated with a modest reduction in blood pressure in a variety of clinical settings, including nondiabetic hypertensive subjects [89]. Taken together, these findings suggest possible additional effects on blood pressure regulation, which are independent of insulin sensitivity, and indeed several lines of evidence suggest that PPAR*γ* may directly regulate vascular tone, for example, through blockade of calcium channel activity in smooth muscle, inhibition of release of endothelin-1, and enhancement of C-type natriuretic peptide release [89].

While no studies of vascular tone or endothelial function have yet been reported in human subjects with PPAR*γ* mutations, mice heterozygous for the equivalent Pro465Leu mutation were found to be hypertensive in the absence of insulin resistance [46]. The hypertension in *Pparg^P465L/+^* mice was associated with increased expression of RAS components in various adipose depots—angiotensinogen (AGT) and angiotensin II receptor subtype 1 (AT1R) in inguinal and gonadal fat, respectively [46]. Interestingly, transgenic mice expressing AGT in adipose tissue have higher BP and increased fat mass [90]. Thus, it is conceivable that modulation of RAS activity in adipose tissue contributes to the decrease in blood pressure, which is seen with TZDs and other PPAR*γ* agonists.

Data relating to differences in blood pressure and Pro12Ala status have proved less informative, with studies again reporting conflicting findings [91, 92], which are likely to reflect other genetic and environmental influences that are at work in the different study populations.

### 3.5. PPAR*γ* and atherosclerosis

Collectively, the individual components of the metabolic syndrome conspire to dramatically increase the risk of cardiovascular disease [93]. PPAR*γ* activation with exogenous ligands such as the TZDs would be predicted to confer significant benefits in this setting, through the amelioration of insulin resistance, dyslipidaemia, and possibly hypertension, albeit at a potential cost of mild weight gain (as a consequence of enhanced adipogenesis and fluid retention). Indeed retrospective human studies have indirectly suggested an atheroprotective effect of TZDs [94], and more recently a prospective trial demonstrated that pioglitazone protected patients with T2DM, albeit modestly, from cardiovascular events [9].

It was therefore surprising and of potential therapeutic concern when Tontonoz et al. reported that PPAR*γ* activation in a premacrophage cell line induced expression of CD36 (also known as FAT—fatty acid translocase), a cellular scavenger receptor for atherogenic low-density lipoprotein (LDL) [95]. Enhanced CD36 expression might be predicted to increase intracellular accumulation of oxidized LDL cholesterol, which could then be catabolized to generate PPAR*γ* ligands (e.g., 9-hydroxyoctadecadienoic acid (9-HODE) and 13-HODE) capable of further receptor activation, thereby creating a vicious feedforward cycle of increasing lipid uptake, and ultimately driving conversion of the macrophage into an atherogenic foam cell [95, 96]. The finding that PPAR*γ* is expressed at relatively high levels in human atherosclerotic plaques further served to fuel concerns [97].

However, almost coincident with these observations, several groups reported that PPAR*γ* ligands could reduce the release of inflammatory cytokines (e.g., TNF-*α* and IL-6) from macrophages, an effect that might be predicted to be antiatherogenic [98, 99]. Several anti-inflammatory mechanisms have been proposed, including inhibition of NF-kB, AP1, and STAT signalling by PPAR*γ* [100].

Subsequent studies have further redressed the balance, with the demonstration that PPAR*γ* ligands exert an opposing effect on SR-A, a second LDL scavenger receptor, downregulating its expression in mouse macrophages [101]. In addition, the nuclear receptor LXR*α* (liver X receptor *α*), which enhances expression of ABCA1 (ATP-binding cassette transporter A1), a protein which mediates cellular cholesterol efflux [102], has also been shown to be a PPAR*γ* target gene in human and mouse macrophages [103, 104]. Taken together, these data suggest a broader spectrum of PPAR*γ* effects within the macrophage with the overall balance favouring cholesterol efflux and an antiatherogenic effect.

At first glance, the finding that only six subjects from a cohort of more than 20 affected PPAR*γ* mutation carriers [18, 23] have documented atheromatous coronary disease might seem surprisingly modest, especially when one considers the severity of insulin resistance, dyslipidaemia, and hypertension found in this group, coupled with the potentially deleterious consequences of dysfunctional PPAR*γ* signalling inside mutant macrophages. However, it is important to note that four of the six affected subjects are/were relatively young females in whom atheromatous coronary disease in the general population is a relatively uncommon occurrence. Accordingly, given that many of the remaining mutation carriers are still relatively young (<50 years), with a predominance of females, it would seem premature to exclude the possibility of accelerated vascular disease in this high-risk group.

There is also an emerging body of epidemiological evidence to suggest an association between the naturally occurring PPAR*γ* polymorphisms and arterial intima media thickness (IMT), and thus indirectly, cardiovascular risk. A study of 154 Japanese T2DM patients found those carrying the Ala12 allele to have a significantly lower carotid IMT than their Pro/Pro counterparts, despite no observed differences in gender, age, fasting blood glucose, lipid profile, or HbA1c [105]. However, differences in BMI and the degree of insulin resistance between the two groups were not reported. Yan et al. used IMT as a secondary outcome measure to investigate the prevalence of the C161T PPAR*γ* polymorphism within 4 different Chinese cohorts; 248 subjects with insulin resistance syndrome (IRS), 163 with essential hypertension, 115 with T2DM and 121 normal controls. They observed that the CC genotype (prevalence 75%) was significantly associated with increased IMT compared to CT and TT genotypes (prevalence 22% and 4%, resp.) within 248 “metabolic syndrome” patients [106]. However interestingly, the prevalence of neither the Pro12Ala nor C161T polymorphism within PPAR*γ* was overrepresented in a large Caucasian cohort (1170 individuals) with angiographically proven coronary heart disease [50], and it is clear that further large-scale studies are needed.

## 4. SELECTIVE PPAR*γ* MODULATION

The ability of TZDs such as rosiglitazone and pioglitazone to enhance insulin sensitivity makes them attractive agents for use in the treatment of T2DM and the metabolic syndrome. Unfortunately however, the initial excitement that followed the introduction of TZDs into clinical practice has been tempered by the realization that for many patients, they afford only modest benefits in terms of glycaemic control—typically lowering glycosylated haemoglobin levels by 1.0%–1.5%—at a cost of weight gain and, in some instances, fluid retention/peripheral oedema [8]. Nevertheless, they represent a “step in the right direction” and have served to emphasize the potential benefits and limitations of modulating PPAR*γ* function in human subjects.

For those seeking to develop the next generation of PPAR*γ* ligands, two (related) key questions must be answered: (1) how much PPAR*γ* activation is desirable, (2) is it possible to separate the receptor's adipogenic actions from those mediating improved insulin sensitivity, that is, to develop selective receptor modulators (so-called SPPARMs) that are capable of regulating glucose and lipid metabolism without promoting adipogenesis. Taking this a step further, if such agents favourably altered receptor function at other sites, for example, within macrophages and the vasculature, then it is conceivable that we might have access to a class of drugs which is almost tailor-made for treating the metabolic syndrome. Precedent for such an approach is provided by raloxifene, a selective oestrogen receptor (ER) modulator (SERM), which is an ER antagonist in breast and endometrium, but an agonist in bone. Examination of the properties of PPAR*γ* in adipocytes suggests that it may be possible to selectively modulate its function in an analogous manner. For example, inside mature adipocytes, certain PPAR*γ* target genes, for example, GyK, require exogenous ligand for activation, while others, for example, aP2, are activated even in the absence of synthetic ligand [24]. The concept of differential modulation of PPAR*γ* activity is also supported by the work of Li and Lazar who have demonstrated that a form of this protein rendered constitutively active by fusion to the powerful VP16 transactivation domain could switch on the adipogenic gene program, yet it was unable to transrepress other PPAR target genes such as that encoding resistin [107].

Promisingly, several groups have independently identified PPAR*γ* ligands with partial agonist activity and only mild/modest effects on adipogenesis, yet with retention of insulin sensitizing properties. MCC-555 is one such compound, whose ability to stimulate PPAR*γ* is highly context-specific [108]. FMOC-L-Leucine, a chemically distinct receptor ligand, whose gene-specific effects appear to reflect differential coactivator recruitment, has been shown to improve insulin sensitivity, yet exert relatively weak adipogenic effects in rodent diabetic models [109]. Similarly, YM440, an analog of the oxadiazolidinediones, improved glycaemic control, but did not alter body fat weight in diabetic db/db mice [110].

The discovery of such compounds has prompted widespread screening of libraries of both structurally related and chemically distinct molecules with the subsequent identification of an array of potential SPPARMs: PAT5a, an unsaturated TZD with partial agonist activity, is a potent antidiabetic agent with only weak adipogenic activity [111]; similar properties have been reported for the novel non-TZD-selective PPAR*γ* modulators nTZDpa [112] and KR-62980 [113]; a panel of N-benzyl-indole-selective PPAR*γ* modulators, with partial agonist activity *in vitro*, exhibited potent glucose-lowering activity in db/db mice, but attenuated increases in heart weight and brown adipose tissue when compared with full agonists [114]. Interestingly, the message that seems to be emerging from these and other similar studies is that ‘activation in moderation’ is the way forward for PPAR*γ*, thus confirming the adage that you can indeed have ‘too much of a good thing.’

## 5. CONCLUSIONS

In just over a decade, PPAR*γ* has evolved from modest beginnings as a simple regulator of adipogenesis to become a key therapeutic target in the fight against the 21st century epidemics of obesity, insulin resistance, and the metabolic syndrome. While pharmacological and animal studies have yielded important information regarding the role of this receptor in the regulation of energy, glucose, and lipid homeostasis, there is little doubt that defining the metabolic consequences associated with polymorphisms and mutations in the human PPAR*γ* gene has contributed significantly to our understanding of the biology of this receptor. Given the significant species-specific differences that exist in metabolism, particularly in relation to lipid homeostasis, it is critical that we continue to identify and study these human experiments of nature, in order to complement the impressive pharmacological and functional genomic approaches that are currently being used to facilitate the development of more superior ligands with enhanced therapeutic impact. Given the apparent inexorable rise in the prevalence of obesity, insulin resistance, and T2DM, the need for such novel therapies could not be more urgent.

## Figures and Tables

**Table 1:** Diagnostic criteria for the human metabolic syndrome. WHO, World Health Organization; EGIR, European Group for the Study of Insulin Resistance; NCEP ATP III, National Cholesterol Education Program Adult Treatment Panel III; IDF, International Diabetes Federation; T2DM, type 2 diabetes mellitus; IGT, impaired glucose tolerance; IR, insulin resistance; TG, triglycerides; HDL, high density lipoprotein cholesterol; BP, blood pressure; BMI, body mass index; WHR, waist hip ratio; WC, waist circumference; AER, albumin excretion rate; M, male; F, female.

WHO, 1999	EGIR, 1999	NCEP ATP III, 2001	IDF, 2005

T2DM or IGT or IR	IR or hyperinsulinaemia, in nondiabetic subjects		*Central obesity:* WC ≥ ethnicity specific cut-offs
with ≥2 of the following	with ≥2 of the following	≥3 of the following	with ≥2 of the following

	*Hyperglycaemia*	*Hyperglycaemia*	*Hyperglycaemia*
	Fasting plasma glucose ≥	Fasting plasma glucose ≥	Fasting plasma glucose ≥
	6.1 mmol/L, but nondiabetic	6.1 mmol/L or	5.6 mmol/L or
		treated with antidiabetic medication.	previously diagnosed T2DM

*Dyslipidaemia*	*Dyslipidaemia*	*Hypertriglyceridaemia*	*Hypertriglyceridaemia*
TG >1.7 mmol/L and/or	TG >2.0 mmol/L or	TG ≥1.7 mmol/L	TG >1.7 mmol/L or
HDL <0.9 mmol/L (M)	HDL <1.0 mmol/L or		treated for this lipid abnormality
HDL <1.0 mmol/L (F)	treated for dyslipidaemia		

		*Low HDL cholesterol*	*Reduced HDL cholesterol*
		HDL <1.0 mmol/L (M)	HDL <1.03 mmol/L (M)
		HDL <1.3 mmol/L (F)	HDL <1.29 mmol/L (F) or
			treated for this lipid abnormality

*Hypertension*	*Hypertension*	*Hypertension*	*Hypertension*
BP ≥140/90 mmHg±medication	BP ≥140/90 mmHg or	BP ≥130/85 mmHg or	BP ≥130/85 mmHg or
	treated for hypertension	treated for hypertension	treated for hypertension

*Obesity*	*Central obesity*	*Central obesity*	*Central obesity*
BMI ≥30 kg/m^2^ or	WC ≥94 cm (M)	WC ≥102 cm (M)	See above—core requirement for
WHR >0.9 (M)	WC ≥80 cm (F)	WC ≥88 cm (F)	diagnosis of syndrome
WHR >0.85 (F)			

*Microalbuminuria*			
Urinary AER >20 mcg/min			
